# Directional and Skill-Level Differences in the Speed–Accuracy Trade-Off During Lacrosse Passing

**DOI:** 10.3390/jfmk11010008

**Published:** 2025-12-25

**Authors:** Saki Tomioka, Hitoshi Koda, Noriyuki Kida

**Affiliations:** 1 Master’s Programs of Applied Biology, Graduate School of Science and Technology, Kyoto Institute of Technology, Hashikami-cho, Matsugasaki, Sakyo-ku, Kyoto 606-8585, Japan; 2 Faculty of Arts and Sciences, Kyoto Institute of Technology, Hashikami-cho, Matsugasaki, Sakyo-ku, Kyoto 606-8585, Japan; h-koda@kit.ac.jp

**Keywords:** Fitts’ law, speed–accuracy tradeoff, consistency, performance analysis

## Abstract

**Background**: Passing in lacrosse is a fundamental skill essential for both offense and defense, directly influencing game flow. Although the speed–accuracy trade-off is well recognized in motor control, its features in lacrosse passing—particularly regarding directional aspects and skill differences—remain unclear. This study quantified the relationship between pass speed, accuracy, bias, and consistency and examined directional effects and skill-level differences. **Methods**: Twenty-two female university players (skilled: *n* = 9; unskilled: *n* = 13) executed overhand passes to a 5 cm × 5 cm target from 11 m under three effort conditions: warm-up, game intensity, and full effort. Ball speed was derived from lateral video, and landing coordinates from posterior footage. Accuracy, bias, and consistency were assessed using radial error (RE), centroid error (CE), absolute CE (|CE|), and bivariate variable error (BVE). Directional patterns were analyzed through lateral and vertical components and the 95% confidence intervals of the major and minor axes of an error ellipse. A two-way analysis of variance was performed with condition as the within-subject factor and skill level as the between-subject factor. **Results**: Ball speed increased significantly across conditions. RE, |CE|, and BVE increased with speed, showing directional dependence: variability expanded mainly along the major axis, while the minor axis remained stable. Skilled players showed smaller RE and BVE, with differences most evident vertically and along the major axis. CE direction stayed consistent, indicating that reduced accuracy stemmed from greater bias magnitude and lower consistency rather than shifts in the mean landing point. **Conclusions**: Findings confirm a speed–accuracy trade-off in lacrosse passing, characterized by directional specificity and skill-related effects. Combining RE, CE, BVE, and ellipse-axis analyses clarified error structure, showing variability concentrated along the movement axis. These results support training focused on vertical control and timing and highlight the value of directional metrics for assessing lacrosse performance. Future research should include male athletes, advanced levels, and in-game scenarios to extend generalizability.

## 1. Introduction

Passing in lacrosse is a fundamental skill that determines possession retention, successful clears, and the creation of scoring opportunities, with its execution directly influencing game flow. Match play in lacrosse is characterized by frequent changes in direction and high-intensity movement demands [1,2], which often constrain the time and space available for skill execution. Under such conditions, players are required to deliver the ball both quickly and accurately within the receiver’s catchable window. Even under relatively stable conditions, however, high-speed passing is often accompanied by reduced control, which can lead to turnovers and delayed offensive transitions. While shooting—another throwing motion in lacrosse—is primarily required of offensive players, passing is essential for both offensive and defensive play. Moreover, errors on the order of only several centimeters can determine the success or failure of an offensive or defensive action. Accordingly, maintaining an appropriate balance between speed and accuracy in passing is tactically critical. Therefore, quantitatively clarifying how passing accuracy and consistency relate to throwing speed is essential for improving lacrosse performance.

In human motor control, the speed–accuracy trade-off—where increasing movement speed leads to greater error—is a well-established principle [3]. This phenomenon has been demonstrated across various tasks such as handball overhand throws, soccer instep kicks, and dart throwing, with studies reporting that accuracy and consistency tend to decrease as initial velocity increases [4,5,6,7]. Conversely, other studies have shown that reducing speed does not necessarily improve accuracy, depending on the sport, skill level, and task design [8,9,10]. Understanding the relationship between speed and accuracy in competitive sports therefore requires consideration of sport-specific and skill-specific factors, highlighting the importance of accumulating fundamental knowledge in this area.

Regarding lacrosse, much of the existing literature has focused on characterizing the physical attributes of lacrosse athletes [11]. Although biomechanical research on shooting motions has increased in recent years [12,13,14,15], studies that quantitatively examine the relationship between speed and accuracy specifically in passing remain limited. Systematic evaluations—including intergroup comparisons between skilled and unskilled players—that assess the relationship between speed and accuracy under multiple effort conditions (warm-up, game intensity, and maximum effort) simulating match situations are particularly scarce.

Although lacrosse passing appears similar to other projectile tasks, its mechanical characteristics—such as bilateral manipulation of a long stick with a long lever arm and an offset release point relative to the body—create constraint conditions that differ from conventional throwing or kicking motions. These sport-specific mechanics may influence how spatial error components respond to increases in movement speed. This approach allows us to test whether classical speed–accuracy principles generalize to lacrosse or whether the sport-specific constraint structure produces distinct error characteristics. Therefore, the present study contributes theoretically by clarifying how task-specific mechanical constraints shape the expression of speed–accuracy trade-offs.

In general, the following two-dimensional spatial evaluation metrics for target-hitting tasks have been widely applied: radial error, defined as the average distance from the target center; centroid error, representing the systematic bias of the landing points relative to the target center; and bivariate variable error, which indicates trial-to-trial consistency [16,17]. These metrics are valuable because they enable separate evaluation of accuracy, systematic bias, and precision. Recent studies have introduced analytical approaches that decompose these metrics into vertical and lateral components or treat the distribution of ball landing points as an ellipse, presenting errors using 95% confidence intervals for the major and minor axes of the error ellipse [18,19]. Although a general tendency has been observed for variability to increase in the primary movement direction during high-speed actions, with stability maintained in the orthogonal direction [20], sport-specific characteristics warrant further examination. Thus, the accumulation of sport-specific evidence remains necessary. To date, no studies have comprehensively evaluated the accuracy, systematic bias, and consistency of lacrosse passes in both vertical and lateral directions while also examining the directional characteristics of error (vertical/lateral) and the distribution shape (95% confidence intervals of the major and minor axes of the error ellipse).

Across many ball sports, skilled players are consistently reported to exhibit smaller errors and lower inter-trial variability than unskilled players [8]. However, there remains insufficient empirical evidence to determine whether similar trends occur in lacrosse passing, or whether skill-related differences in error characteristics are more pronounced in the vertical or lateral directions. Considering constraints specific to the sport—such as the unique shape and handling properties of the lacrosse stick and the spatial range within which the receiver can effectively respond during the catching motion—different directional error characteristics may emerge.

Given this background, the present study aimed to clarify the relationship between pass speed and accuracy in lacrosse, as well as its directional and skill-related characteristics. Specifically, we sought to examine the effects of increased pass speed on accuracy (radial error, RE), systematic bias (centroid error, CE; absolute centroid error, |CE|), and consistency (bivariate variable error, BVE), and to determine how these effects vary by skill level and directional component of error (vertical/lateral or along the major/minor axes).

To achieve this objective, female university lacrosse players were instructed to pass toward a fixed target from a set distance under three effort conditions: warm-up, game intensity, and full effort. Multivariate analyses were conducted using initial ball speed, performance variables (radial error, RE; centroid error, CE; bivariate variable error, BVE), and the characteristics of the error ellipse (95% confidence intervals for the major and minor axes). In addition, comparisons between skilled and unskilled groups were performed to determine whether the trade-offs observed between increased speed and elevated RE, CE, or BVE were reproduced in passing actions; whether these effects or skill differences manifested directionally (e.g., vertical component dominance or long-axis selection); and whether separating CE into its direction (sign) and magnitude (|CE|) could distinguish whether the decline in accuracy with increased speed was due to systematic shifts in the mean landing position or increases in positional bias and dispersion. By applying component-specific evaluations (vertical/lateral) and ellipsoidal axis analyses to the three performance metrics (RE, CE, and BVE), we aimed to visualize the distribution and directional structure of passing errors, thereby advancing sport-specific understanding of the speed–accuracy relationship in lacrosse.

## 2. Materials and Methods

### 2.1. Participants

The participants were 22 female university students (age: 20 ± 2 years) with lacrosse experience ranging from 3 months to 6 years. All participants were able to perform passing and catching without requiring technical instruction. In this study, the skilled group was defined as players who had at least two consecutive years of experience as starting members in the Kansai Student Lacrosse League. Based on this criterion, 9 players were categorized into the skilled group and 13 players into the unskilled group. Exclusion criteria included a history of orthopedic disorders or any movement restrictions or pain during the experimental tasks; no participants were excluded. The experimental procedure was explained to all participants in advance and reconfirmed on the day of testing. The study was conducted after obtaining written informed consent from each participant and was approved by the Kyoto Institute of Technology Ethics Committee for Scientific Research Involving Human Subjects (Approval No. 2023-65).

### 2.2. Experimental Task

**Figure 1:**
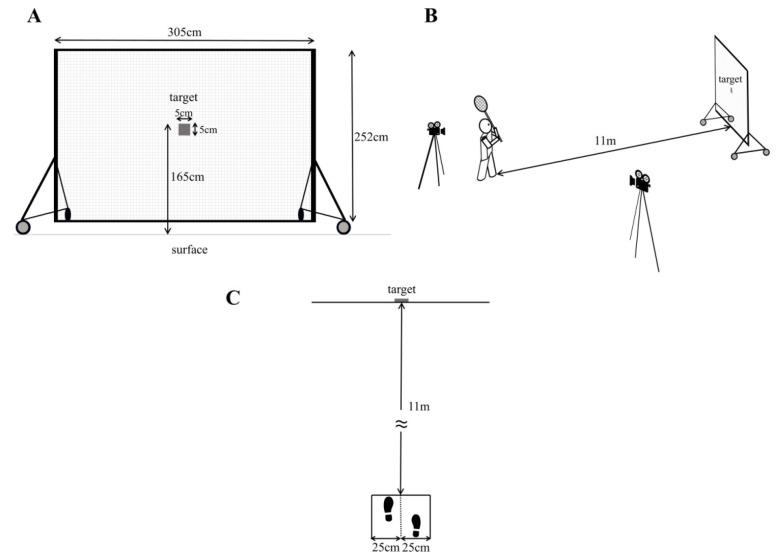
Experimental setup. (**A**) The target (5 cm × 5 cm) was marked with tape on a net (2.52 m high × 3.05 m wide), and visibility was confirmed for all participants. (**B**) The throwing distance was fixed at 11 m. (**C**) Participants stood facing the target with their non-dominant foot forward and were instructed to maintain the front foot within ±25 cm of the center line.

The experiment was conducted on an outdoor lacrosse field. After completing a sufficient self-paced warm-up, participants performed the passing task using a regulation lacrosse stick (cross; compliant with Japan Lacrosse Association standards) and a certified lacrosse ball (Wolf Athletics; NOCSAE standard, SEI certified). The target was marked with tape (5 cm × 5 cm) on a net (2.52 m in height × 3.05 m in width), and visibility was confirmed for all participants (Figure 1A). The center of the target was positioned 1.65 m above the ground, corresponding to the approximate height of a receiver’s stick face.

The throwing distance was set to 11 m (Figure 1B), corresponding to the Japan Lacrosse Association’s free-shot distance (as of 2024). This distance represented a typical range for direct passes—those that reach the receiver without bouncing—and was verified through preliminary testing to reflect realistic in-game conditions.

Participants stood facing the target with their non-dominant foot forward and were instructed to maintain the front foot within ±25 cm of the center line (Figure 1C). Passes were executed using a standard overhand passing motion intended for a teammate.

Three effort conditions were examined: warm-up, game intensity, and full effort. Participants were instructed to perform each condition at a self-selected pace. In the warm-up condition, participants performed relaxed, routine passes for preparation. In the game-intensity condition, they executed fast and accurate passes as if under competitive pressure. In the full-effort condition, participants attempted passes at their maximum possible speed, without regard for accuracy.

Each participant completed 30 trials in total (10 warm-up, 15 game-intensity, and 5 full-effort) in that order, with approximately 3 min of rest between conditions. The number of trials was determined based on previous findings indicating that 8–12 repetitions are required for the stabilization of movement characteristics [21] while also minimizing fatigue and the risk of injury during maximal-effort tasks. The number of full-effort trials was limited to five for safety reasons. The number of repetitions differed between conditions because the full-effort condition involved greater physical load, and limiting the total number reduced fatigue-related decline in performance. Although unequal repetitions may influence statistical stability, the same analysis procedure was applied across conditions, and the number of trials per condition remained within the range commonly used in motor performance research.

Video recordings were obtained using two digital cameras (JVC GC-YJ40; Victor Company of Japan, Limited; Yokohama, Japan; 59.94 fps): one positioned laterally (approximately 5 m from the throwing side) to capture the throwing motion, and the other positioned posteriorly (approximately 1.7 m behind the target) to record the ball impact locations. All recordings were conducted outdoors under natural sunlight, which provided stable and uniform lighting.

### 2.3. Data Acquisition

Coordinates were obtained from the lateral and posterior video images using the two-dimensional Direct Linear Transformation method. Coordinate measurements were performed using Frame-DIAS V software (version 2.33, Q’s fix). Calibration for the lateral images was conducted with a 2 m calibration rod and a leveling device, referencing four points that defined a 2 m square on the participants’ sagittal plane. For the posterior images, four points on the target net (252 cm × 305 cm) were used for calibration. The coordinate system was defined with the target center as the origin (0, 0), where the rightward and upward directions were assigned as positive axes.

**Figure 2:**
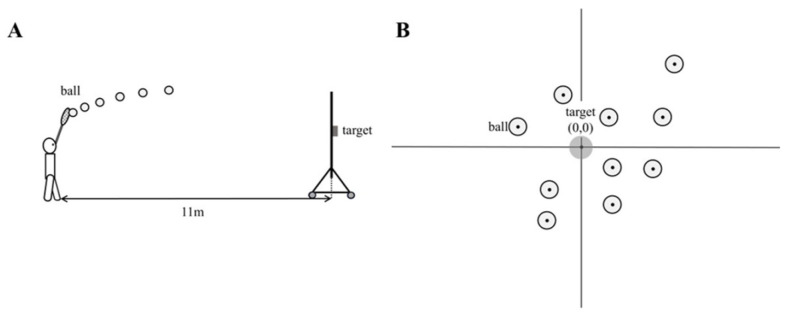
Coordinate measurement procedure. (**A**) Ball coordinates were extracted from lateral footage for six frames following release to calculate the mean ball speed. (**B**) Landing coordinates relative to the target were measured from posterior footage, with net movement indicating the moment of ball arrival.

From the lateral footage, the ball’s x- and y-coordinates were digitized for six consecutive frames beginning at the moment the ball left the stick (Figure 2A), and the average ball speed was calculated from the displacement across those frames. This window was selected because pilot testing demonstrated that using six frames minimized digitization noise and produced more stable velocity estimates compared with shorter intervals (e.g., three frames) From the posterior footage, the coordinates of the ball’s landing point relative to the target center were measured (Figure 2B). The frame in which the ball caused the target net to move was defined as the moment of impact.

To verify the reliability of the coordinate digitization procedure, ten throws from the same participant under the same condition were digitized three times, and intraclass correlation coefficients (ICC, single measures, two-way random, absolute agreement) were calculated. The results indicated excellent reliability for both coordinates, with ICC = 1.000 (95% CI: 0.999–1.000) for the x-coordinate and ICC = 0.989 (95% CI: 0.970–0.997) for the y-coordinate.

### 2.4. Error Metrics and Computation

Using the obtained coordinate data, the distance between the target center and each ball’s impact point was calculated, and its relationship with effort condition and ball speed was examined.

**Figure 3:**
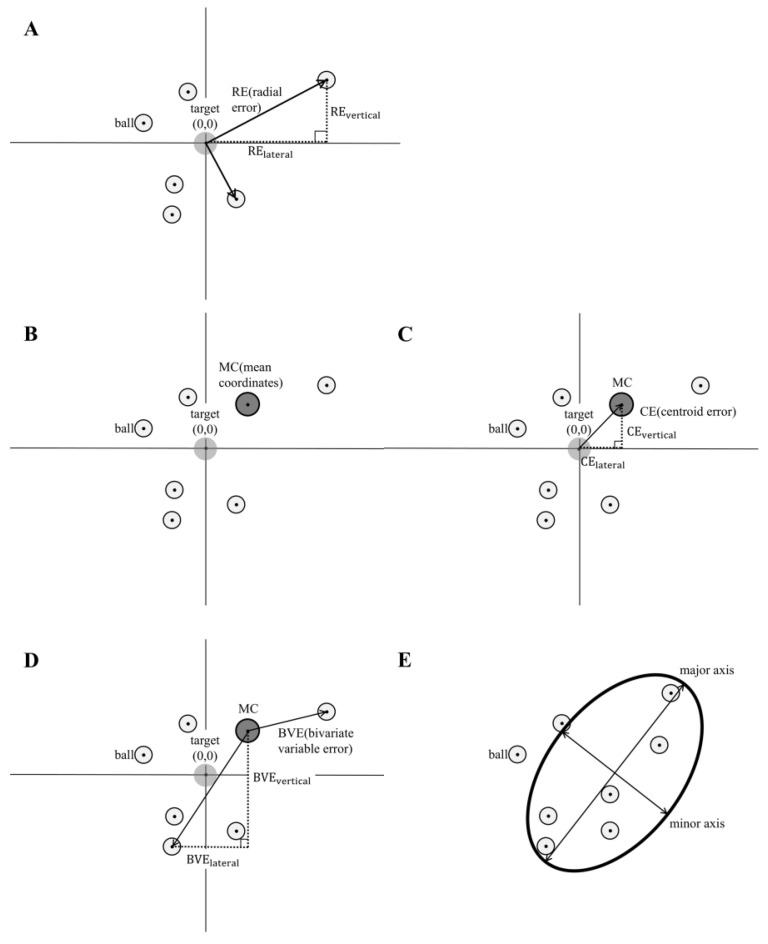
Definitions of performance indices. (**A**) Radial error (RE) represents accuracy, indicating the average distance of the ball from the target center. (**B**) Mean coordinates (MC) represent the average landing position of each participant’s throws, serving as the center of the individual landing-point distribution. (**C**) Centroid error (CE) represents systematic bias, showing the deviation of the mean landing point from the target center. (**D**) Bivariate variable error (BVE) represents consistency, reflecting the variability of landing points across trials. (**E**) Landing points for each participant were approximated by an ellipse, and the lengths of the major and minor axes were calculated using 95% confidence intervals.

The mean radial error, referred to as RE, was defined as the average distance from the target center to the ball’s impact point. It was calculated using the x- and y-coordinates of the i-th trial as follows (Figure 3A), where n represents the number of passes per condition:

$$RE=\frac{1}{n}\sum_{i=1}^{n}\sqrt{{x_{i}}^{2}+{y_{i}}^{2}}$$

$${RE}_{lateral}=\frac{1}{n}\sum_{i=1}^{n}\left|x_{i}\right|$$

$${RE}_{vertical}=\frac{1}{n}\sum_{i=1}^{n}\left|y_{i}\right|$$

The mean coordinates (MC) were defined as the average of the ball’s impact-point coordinates across all trials for each condition and each participant (Figure 3B):

$$MC=(\bar{x},\bar{y})=(\frac{1}{n}\sum_{i=1}^{n}x_{i},\frac{1}{n}\sum_{i=1}^{n}y_{i})$$

The centroid error, referred to as CE, was defined as the distance between the movement center and the target center (Figure 3C). The signed CE indicated the direction of systematic bias, whereas the absolute value of CE represented the magnitude of that bias:

$${CE}_{lateral}=\bar{x}$$

$${CE}_{vertical}=\bar{y}$$

$${|CE}_{lateral}|=\left|\bar{x}\right|$$

$${|CE}_{vertical}|=|\bar{y|}$$

The bivariate variable error, referred to as BVE, was defined as the mean distance between the movement center and each ball’s impact point (Figure 3D):

$$BVE=\frac{1}{n}\sum_{i=1}^{n}\sqrt{{\left(x_{i}-\bar{x}\right)}^{2}+{\left(y_{i}-\bar{y}\right)}^{2}}$$

$${BVE}_{lateral}=\frac{1}{n}\sum_{i=1}^{n}|x_{i}-\bar{x}|$$

$${BVE}_{vertical}=\frac{1}{n}\sum_{i=1}^{n}|y_{i}-\bar{y}|$$

Furthermore, the lengths of the 95% confidence intervals (CIs) for the major and minor axes of the error ellipse were calculated. Principal component analysis was applied to the distribution of the ball impact points, and the resulting data were approximated by an ellipse (Figure 3E).

### 2.5. Statistical Analysis

A two-way analysis of variance (ANOVA) was conducted with the performance variables—RE, CE, and BVE—as dependent variables. Effort condition was treated as a within-subject factor, and skill group (skilled vs. unskilled) was treated as a between-subject factor. The level of statistical significance was set at *p* < 0.05. All analyses were performed using IBM SPSS Statistics (version 26; IBM Corp., Armonk, NY, USA).

## 3. Results

### 3.1. Ball Speed and Distance from Target (Radial Error)

Table 1 presents the mean and standard deviation of ball speed and RE by skill level and effort condition. The two-way ANOVA with skill level and condition as factors revealed that, for initial ball speed, neither the interaction effect nor the main effect of skill group was significant. However, the main effect of condition was significant (*F*_2, 40_ = 130.995, *p* < 0.001, *η*^2^ = 0.868). Ball speed was highest in the full-effort condition, followed by the game-intensity condition and then the warm-up condition. Post hoc multiple comparisons indicated significant differences among all conditions.

No significant interaction effect was observed for RE (*F*_2, 40_ = 1.418, *p* = 0.254, *η*^2^ = 0.066). A significant main effect of skill level was found (*F*_1, 20_ = 13.887, *p* = 0.001, *η*^2^ = 0.410), with the skilled group exhibiting smaller RE values than the unskilled group. A significant main effect of condition was also observed (*F*_2, 40_ = 21.420, *p* < 0.001, *η*^2^ = 0.517). Post hoc multiple comparisons indicated significantly larger RE values in the full-effort condition than in both the warm-up and game-intensity conditions. When RE was examined separately for the vertical and lateral components, no significant group-by-condition interaction was found for either component (${RE}_{lateral}$, *F*_2, 40_ = 0.981, *p* = 0.384, *η*^2^ = 0.047; ${RE}_{vertical}$, *F*_2, 40_ = 0.881, *p* = 0.422, *η*^2^ = 0.042). Regarding the main effect of skill level, it was not significant for the lateral component but was significant for the vertical component (${RE}_{lateral}$, *F*_1, 20_ = 2.990, *p* = 0.099, *η*^2^ = 0.130; ${RE}_{vertical}$, *F*_1, 20_ = 12.204, *p* = 0.002, *η*^2^ = 0.379), with the skilled group demonstrating significantly smaller vertical RE values than the unskilled group. The main effect of condition was significant for both components (${RE}_{lateral}$, *F*_2, 40_ = 6.212, *p* = 0.004, *η*^2^ = 0.237; ${RE}_{vertical}$, *F*_2, 40_ = 16.943, *p* < 0.001, *η*^2^ = 0.459). Post hoc comparisons revealed significantly larger ${RE}_{lateral}$ values in the full-effort condition compared with the warm-up condition, and significantly larger ${RE}_{vertical}$ values in the full-effort condition compared with both the warm-up and game-intensity conditions.

### 3.2. Passing Center Coordinate (Centroid Error)

Table 2 presents the mean and standard deviation of CE by skill level and effort condition. For the lateral component (${CE}_{lateral}$), a significant interaction between skill level and condition was found (*F*_2, 40_ = 3.948, *p* = 0.027, *η*^2^ = 0.165); however, the simple main effects were not significant. For the vertical component (${CE}_{vertical}$), no significant interaction was observed (*F*_2, 40_ = 0.444, *p* = 0.645, *η*^2^ = 0.022), and neither the main effect of skill group nor that of condition was significant (*F*_1, 20_ = 0.068, *p* = 0.797, *η*^2^ = 0.003; *F*_2, 40_ = 0.112, *p* = 0.894, *η*^2^ = 0.006, respectively).

For the absolute values of CE, no significant interaction was found for either $|{CE}_{lateral}$| or $|{CE}_{vertical}$| $|{CE}_{lateral}$|, *F*_2, 40_ = 0.895, *p* = 0.417, *η*^2^ = 0.043; $|{CE}_{vertical}$|, *F*_2, 40_ = 0.765, *p* < 0.472, *η*^2^ = 0.037). The main effect of skill level was not significant for $\left|{CE}_{lateral}\right|$ (*F*_1, 20_ = 2.434, *p* = 0.134, *η*^2^ = 0.108) but was significant for $|{CE}_{vertical}$| (*F*_1, 20_ = 4.868, *p* = 0.039, *η*^2^ = 0.196), with the skilled group showing smaller $|{CE}_{vertical}$| values than the unskilled group. Significant main effects of condition were observed for both components (${|CE}_{lateral}|$, *F*_2, 40_ = 4.481, *p* = 0.018, *η*^2^ = 0.183; $|{CE}_{vertical}$|, *F*_2, 40_ = 11.236, *p* < 0.001, *η*^2^ = 0.360). Post hoc multiple comparisons revealed that values were significantly larger in the full-effort condition than in the warm-up condition for both $\left|{CE}_{lateral}\right|$ and $|{CE}_{vertical}$|.

### 3.3. Passing Consistency (Bivariate Variable Error) and Distribution Shape Metrics

Table 3 presents the mean and standard deviation of BVE and distribution shape indices by skill level and effort condition. For overall BVE, no significant interaction effect was observed (*F*_2, 40_ = 0.554, *p* = 0.579, *η*^2^ = 0.027). The main effect of skill level was significant (*F*_1, 20_ = 8.369, *p* = 0.009, *η*^2^ = 0.295), with the skilled group exhibiting smaller BVE values than the unskilled group. Additionally, the main effect of condition was significant (*F*_2, 40_ = 7.600, *p* = 0.002, *η*^2^ = 0.275), with significantly larger values observed in the full-effort condition than in the warm-up condition.

When BVE was examined separately for the lateral and vertical components, no significant interaction effects were found for either component (${BVE}_{lateral}$, *F*_2, 40_ = 0.251, *p* = 0.779, *η*^2^ = 0.012; ${BVE}_{vertical}$, *F*_2, 40_ = 0.683, *p* = 0.511, *η*^2^ = 0.033). The main effect of skill level was not significant for ${BVE}_{lateral}$ (*F*_1, 20_ = 1.449, *p* = 0.243, *η*^2^ = 0.068) but was significant for ${BVE}_{vertical}$ (*F*_1, 20_ = 12.117, *p* = 0.002, *η*^2^ = 0.377), with the unskilled group showing significantly larger values than the skilled group. Furthermore, the main effect of condition was significant for both components, and post hoc multiple comparisons revealed that the full-effort condition produced significantly larger BVE values than the warm-up condition.

The passing distribution was modeled as an ellipse, and 95% CIs were calculated for its major and minor axes. Two-way ANOVA results revealed no significant interaction effects for either axis. The main effect of skill level was not significant for the minor axis but was significant for the major axis, with the skilled group exhibiting significantly smaller values than the unskilled group. Furthermore, the main effect of condition was significant only for the major axis, with the full-effort condition producing significantly larger values than both the warm-up and game-intensity conditions.

## 4. Discussion

### 4.1. Comparison of Passing Conditions

In this study, increases in initial ball speed across the effort conditions (warm-up, game-intensity, and full-effort) resulted in significant increases in RE and BVE, with the highest values observed in the full-effort condition. These findings support the classical speed–accuracy trade-off [3] and are consistent with observations in handball [22], soccer [6], and darts [5]. CE showed minimal directional change across conditions, while its absolute value (|CE|) increased with speed, indicating that high-velocity passes primarily enlarge the magnitude of deviation and reduce consistency rather than shift the mean landing position.

Analyses of the vertical and lateral components of RE and BVE revealed clear directional specificity. This pattern was further confirmed by the behavior of the major and minor axes of the error ellipse. Variability increased selectively along the major axis as speed increased, whereas the minor axis remained unchanged. This pattern reflects a general motor-control tendency in which variability grows in the primary movement direction but remains stable in the orthogonal direction [20]. Differences in the lateral component can partially be explained by the projection of the tilted major axis onto the coordinate system, rather than genuine increases in lateral variability. Because CE direction remained stable across conditions, the observed reduction in accuracy is best attributed to increases in deviation magnitude and decreased consistency along the major axis.

The selective expansion of the major axis under full-effort conditions may result from increased release-timing variability and impulse-adjustment noise at higher speeds, while the minor axis remains structurally constrained by stick mechanics, body configuration, and visual–postural feedback. This interpretation aligns with previous work demonstrating that variability increases primarily along the long axis of movement [20,23].

Comparisons between the warm-up and game-intensity conditions showed small increases in |RE|, |CE|, and BVE, with only limited change in the major axis and no change in the minor axis, indicating that differences in this speed range arise mainly from increases in bias magnitude and dispersion. In contrast, from the game-intensity to full-effort conditions, |RE|, |CE|, and BVE increased markedly, accompanied by significant expansion of the major axis but not the minor axis. Vertical-direction indices increased substantially, while lateral differences were largely attributable to projection effects. Because CE did not shift directionally, reductions in accuracy at maximal effort stemmed from enlarged variability and decreased inter-trial consistency along the major axis.

Taken together, these results demonstrate clear directional specificity in the speed–accuracy relationship in lacrosse passing: variability increases predominantly along the major axis, whereas the minor axis remains stable. Evaluating only vertical and lateral components makes it difficult to disentangle projection effects from actual component-specific differences, but incorporating both ellipse axes enabled separation of these influences. The combined use of RE, CE, BVE, and ellipse-axis indices therefore provided an effective framework for visualizing direction-dependent declines in accuracy and clarifying the underlying components of performance loss.

### 4.2. Comparison Between Skill Levels

The interaction between skill level and condition was significant only for the lateral component of centroid error (${CE}_{lateral})$, with no other effects reaching statistical significance. In between-group comparisons, the skilled group consistently exhibited smaller RE and BVE than the unskilled group, indicating superior passing accuracy and inter-trial consistency. These findings align with previous research on throwing, kicking, and target-hitting tasks [4,8], and the present study extends this general pattern to lacrosse passing, a movement frequently executed during gameplay.

A clear directional dependence associated with skill level also emerged. Although group differences were minimal for the lateral component and for the minor axis of the error ellipse, the unskilled group showed substantially greater errors in the vertical component and along the major axis. The limited differences in lateral errors and minor-axis variability may reflect the physical constraints imposed by stick manipulation and trunk rotation, which structurally suppress lateral fluctuations. In contrast, vertical control relies heavily on release timing and fine impulse adjustments, making it more susceptible to residual variability during earlier learning stages. Consistent with this interpretation, the skilled group demonstrated reduced vertical error and variability, suggesting refinement in timing control gained through skill acquisition.

Some of the observed differences in the lateral component may stem from the projection of the distribution’s primary direction (i.e., the tilted major axis) onto the coordinate axes. By integrating component-specific indices with ellipse-based approximations, the orientation and geometry of the error structure could be clearly distinguished, allowing for a more accurate interpretation of skill-dependent error characteristics.

### 4.3. Practical Implications for Coaching

From a coaching perspective, several practical implications emerge from the present findings. In lacrosse instruction, a fundamental technical emphasis is placed on maintaining a vertical stick swing to stabilize lateral control, as lateral deviations are more difficult for receivers to compensate for than vertical deviations. This emphasis likely contributes to the relatively small lateral variability seen even among less-skilled players, whereas greater vertical variability may persist due to underdeveloped control of release timing and stick height.

The directional increase in error observed under full-effort conditions further suggests that training should specifically target stability along the major-axis direction, particularly in the vertical component where novice players exhibited pronounced variability. Drills that focus on consistent release timing, controlled impulse generation, and progressively higher-speed passing may help players maintain accuracy as intensity increases. Because lateral variability remains relatively stable even at higher speeds, coaches may prioritize correcting vertical deviations when aiming to improve overall passing accuracy.

Together, these insights indicate that refined timing control is a key characteristic of skilled performers. Therefore, incorporating feedback-based practice, video-assisted correction, or constraints-led drills may accelerate the acquisition of vertical stability and overall accuracy in developing players [24].

### 4.4. Significance, Novelty, Limitations, and Future Directions

The methodological novelty of this study lies in the integrated application of four complementary performance indices: RE, CE, BVE, and the axes of the error ellipse. By assessing overall accuracy through RE, directional bias via CE, bias magnitude through |CE|, consistency through BVE, and the directional characteristics of variability via the ellipse axes, the present study provided a multifaceted evaluation of lacrosse passing accuracy. In particular, the incorporation of ellipse-axis analysis revealed that the increase in variability accompanying higher speeds was direction-specific, occurring predominantly along the major axis. Furthermore, it demonstrated that the influence of skill level on variability differs between directions that exert substantial versus limited effects on passing performance. These findings represent both a methodological and theoretical advancement beyond previous studies—which primarily relied on mean distance-based metrics—by elucidating the directional structure of error distributions and clarifying the distinct contributions of individual components.

However, this study has several limitations. First, the participants were limited to female collegiate lacrosse players; therefore, caution is warranted when generalizing the findings to male players or athletes at different competitive levels. Moreover, the present experiment included players belonging to the Kansai Lacrosse League Division II. The mean ball speeds under the full-effort condition were 65.3 ± 6.3 km/h for the unskilled group and 66.9 ± 7.7 km/h for the skilled group—values comparable to those reported for NCAA Division III players in a previous study [9]. Hence, caution should be exercised when inferring the characteristics of pass control among players competing at higher levels based on these results. Second, the experiment was conducted under controlled conditions in terms of target distance and trial environment, which may not fully replicate the dynamic and pressured situations encountered during actual gameplay. Game-related constraints such as in-game decision-making demands and opponent pressure may further influence passing accuracy [1]. Factors such as task variability may also affect performance; however, these effects could not be examined within the current experimental design. Third, the conditions were performed in a fixed order to ensure adequate warm-up before maximal-effort trials, which may have introduced practice or fatigue effects. Additionally, the unequal number of trials across conditions may have influenced the stability of consistency-related metrics such as BVE and ellipse axes. Future studies should use counterbalanced orders and equalized trial counts, or apply robust estimation methods (e.g., bootstrapping, hierarchical Bayesian or mixed-effects models) to improve statistical reliability.

Future research should also broaden the participant pool to include athletes of varying skill levels and both genders, and incorporate motion capture and electromyography to elucidate the neuromechanical mechanisms underlying the direction-dependent increase in variability at higher throwing speeds. Furthermore, examining passing actions under competitive match conditions and one-on-one scenarios would enable verification of the control strategies used to maintain accuracy and consistency under realistic performance constraints. Such investigations will further enhance the applicability and practical value of the multifaceted evaluation framework proposed in the present study.

## Figures and Tables

**Table 1:** Ball speed and distance from target (RE).

		Warm-Up	Game-Intensity	Full-Effort		Main Effect		Interaction
						Group	Condition	
ball speed (km/h)								
Unskilled group	M ± SD	52.5 ± 4.9	57.8 ± 4.5	65.3 ± 6.3	*F*	0.353	130.995	0.710
	95%CI	[49.9, 55.1]	[55.0, 60.5]	[61.3, 69.3]	*p*	0.559	<0.001	0.498
Skilled group	M ± SD	52.7 ± 3.7	59.9 ± 5.1	66.9 ± 7.7	*η* ^2^	0.017	0.868	0.034
	95%CI	[49.6, 55.8]	[56.6, 63.2]	[62.1, 71.7]				
*RE* (cm)								
Unskilled group	M ± SD	36.1 ± 12.9	44.9 ± 12.7	60.0 ± 11.4	*F*	13.887	21.420	1.418
	95%CI	[30.1, 42.1]	[38.5, 51.3]	[52.1, 68.0]	*p*	0.001	<0.001	0.254
Skilled group	M ± SD	27.7 ± 4.6	29.3 ± 8.0	41.8 ± 16.6	*η* ^2^	0.410	0.517	0.066
	95%CI	[20.4, 34.9]	[21.6, 37.0]	[32.2, 51.4]				
*RE lateral* (cm)								
Unskilled group	M ± SD	19.6 ± 9.8	26.3 ± 14.8	34.7 ± 19.9	*F*	2.990	6.212	0.981
	95%CI	[14.5, 24.6]	[19.2, 33.4]	[24.2, 45.3]	*p*	0.099	0.004	0.384
Skilled group	M ± SD	16.5 ± 6.6	16.7 ± 6.9	23.2 ± 15.6	*η* ^2^	0.130	0.237	0.047
	95%CI	[10.5, 22.6]	[8.2, 25.2]	[10.5, 35.9]				
*RE vertical* (cm)								
Unskilled group	M ± SD	26.0 ± 9.7	31.3 ± 6.5	45.4 ± 18.1	*F*	12.204	16.943	0.881
	95%CI	[21.5, 30.5]	[27.7, 34.8]	[36.6, 54.3]	*p*	0.002	<0.001	0.422
Skilled group	M ± SD	18.6 ± 3.1	20.0 ± 5.7	30.5 ± 10.1	*η* ^2^	0.379	0.459	0.042
	95%CI	[13.2, 24.0]	[15.7, 24.3]	[19.8, 41.2]				

Notes. Values are presented as mean ± standard deviation, followed by 95% confidence intervals (95% CI). *RE* = radial error; ${RE}_{lateral}$ and ${RE}_{vertical}$ = lateral and vertical components of radial error. *η*^2^ = partial eta squared. Ball speed is expressed in km/h; error measures are expressed in cm.

**Table 2:** The passing center coordinates (CE).

		Warm-Up	Game-Intensity	Full-Effort		Main Effect		Interaction
						Group	Condition	
*CE lateral* (cm)								
Unskilled group	M ± SD	1.8 ± 16.6	1.0 ± 24.5	−15.3 ± 34.1	*F*	1.081	0.222	3.948
	95%CI	[−6.6, 10.3]	[−10.9, 12.8]	[−32.4, 1.9]	*p*	0.311	0.802	0.027
Skilled group	M ± SD	0.3 ± 10.8	−0.3 ± 12.0	10.4 ± 21.5	*η* ^2^	0.051	0.011	0.165
	95%CI	[−9.8, 10.4]	[−14.5, 13.9]	[−10.2, 31.0]				
*CE vertical* (cm)								
Unskilled group	M ± SD	9.8 ± 19.0	1.3 ± 17.3	5.2 ± 42.2	*F*	0.068	0.112	0.444
	95%CI	[0.3, 19.4]	[−7.2, 9.7]	[−15.7, 26.2]	*p*	0.797	0.894	0.645
Skilled group	M ± SD	3.0 ± 12.0	7.0 ± 9.4	1.0 ± 24.6	*η* ^2^	0.003	0.006	0.022
	95%CI	[−8.5, 14.5]	[−3.2, 17.2]	[−24.1, 26.2]				
*|CE lateral|* (cm)								
Unskilled group	M ± SD	12.7 ± 10.3	14.5 ± 19.4	28.2 ± 23.5	*F*	2.434	4.481	0.895
	95%CI	[7.4, 17.9]	[5.3, 23.8]	[15.5, 40.9]	*p*	0.134	0.018	0.417
Skilled group	M ± SD	8.0 ± 6.7	7.8 ± 8.7	13.6 ± 19.4	*η* ^2^	0.108	0.183	0.043
	95%CI	[1.7, 14.3]	[−3.3, 18.9]	[−1.7, 28.8]				
*|CE vertical|* (cm)								
Unskilled group	M ± SD	17.7 ± 11.2	12.9 ± 11.0	34.6 ± 22.6	*F*	4.868	11.236	0.765
	95%CI	[12.4, 23.1]	[7.3, 18.5]	[23.9, 45.3]	*p*	0.039	<0.001	0.472
Skilled group	M ± SD	10.5 ± 5.5	9.1 ± 7.2	21.5 ± 9.4	*η* ^2^	0.196	0.360	0.037
	95%CI	[4.1, 17.0]	[2.4, 15.8]	[8.6, 34.3]				

Notes. Values are presented as mean ± standard deviation, followed by 95% confidence intervals (95% CI). *CE* = centroid error; ${CE}_{lateral}$ and ${CE}_{vertical}$ = lateral and vertical components of centroid error. ${|CE}_{lateral}|$ and $\left|{CE}_{vertical}\right|$ vertical represent the absolute magnitudes of lateral and vertical centroid errors. *η*^2^ = partial eta squared. All error values are expressed in centimeters.

**Table 3:** The linear distance from the passing center (BVE) and the 95%CIs for the major and minor axes of the ellipse.

		Warm-Up	Game-Intensity	Full-Effort		Main Effect		Interaction
						Group	Condition	
*BVE* (cm)								
Unskilled group	M ± SD	30.7 ± 9.9	37.3 ± 7.1	41.4 ± 13.8	*F*	8.369	7.600	0.554
	95%CI	[25.8, 35.7]	[33.3, 41.3]	[34.0, 48.7]	*p*	0.009	0.002	0.579
Skilled group	M ± SD	24.1 ± 5.8	25.8 ± 6.6	31.6 ± 10.7	*η* ^2^	0.295	0.275	0.027
	95%CI	[18.2, 30.0]	[21.0, 30.6]	[22.8, 40.4]				
*BVE lateral* (cm)								
Unskilled group	M ± SD	16.8 ± 7.6	19.3 ± 5.8	21.4 ± 8.9	*F*	1.449	4.482	0.251
	95%CI	[12.3, 21.3]	[16.2, 22.3]	[16.7, 26.2]	*p*	0.243	0.018	0.779
Skilled group	M ± SD	14.4 ± 8.1	14.9 ± 4.4	19.2 ± 7.0	*η* ^2^	0.068	0.183	0.012
	95%CI	[8.9, 19.8]	[11.3, 18.6]	[13.5, 24.8]				
*BVE vertical* (cm)								
Unskilled group	M ± SD	22.0 ± 8.3	28.6 ± 5.6	32.3 ± 14.0	*F*	12.117	4.798	0.683
	95%CI	[18.0, 26.1]	[25.4, 31.8]	[25.1, 39.5]	*p*	0.002	0.014	0.511
Skilled group	M ± SD	16.4 ± 4.5	18.2 ± 5.3	21.4 ± 9.6	*η* ^2^	0.377	0.193	0.033
	95%CI	[11.5, 21.3]	[14.4, 22.0]	[12.8, 30.1]				
Minor axis (cm)								
Unskilled group	M ± SD	38.5 ± 14.4	43.6 ± 12.6	37.3 ± 19.4	*F*	0.763	1.147	1.135
	95%CI	[30.1, 46.9]	[37.0, 50.2]	[26.7, 47.9]	*p*	0.393	0.328	0.332
Skilled group	M ± SD	30.8 ± 14.7	36.2 ± 9.5	38.9 ± 16.8	*η* ^2^	0.037	0.054	0.054
	95%CI	[20.8, 40.9]	[28.2, 44.1]	[26.1, 51.7]				
Major axis (cm)								
Unskilled group	M ± SD	77.8 ± 24.9	97.1 ± 19.8	120.6 ± 37.7	*F*	13.131	12.643	1.035
	95%CI	[65.6, 90.1]	[86.6, 107.6]	[100.7, 140.4]	*p*	0.002	<0.001	0.364
Skilled group	M ± SD	60.9 ± 13.6	64.6 ± 15.5	85.5 ± 28.2	*η* ^2^	0.396	0.387	0.049
	95%CI	[46.1, 75.6]	[52.0 77.2]	[61.6, 109.3]				

Notes. Values are presented as mean ± standard deviation, followed by 95% confidence intervals (95% CI). *BVE* = bivariate variable error; ${BVE}_{lateral}$ and ${BVE}_{vertical}$ = lateral and vertical components of bivariate variable error. “Major axis” and “Minor axis” represent the lengths of the 95% confidence intervals of the major and minor axes of the error ellipse obtained from principal component analysis of the landing-point distribution. All error values are expressed in centimeters. *η*^2^ = partial eta squared.

## Data Availability

The original contributions presented in this study are included in the article. Further inquiries can be directed to the corresponding author.

## Abbreviations

The following abbreviations are used in this manuscript:

RE	Radial error
CE	Centroid error
BVE	Bivariate variable error
